# A Simple Technique Using Peri-Prosthetic Irrigation Improves Implant Salvage Rates in Immediate Implant-Based Breast Reconstruction

**DOI:** 10.3390/medicina59112039

**Published:** 2023-11-19

**Authors:** Manoj Srinivas Gowda, Sadaf Jafferbhoy, Sekhar Marla, Sankaran Narayanan, Soni Soumian

**Affiliations:** 1 Department of General Surgery, James Cook University Hospital, Middlesbrough TS4 3BW, UK; manoj.gowda@nhs.net; 2 Department of Breast Surgery, University Hospitals of North Midlands, Stoke-on-Trent ST4 6QG, UK; sadaf.jafferbhoy@uhnm.nhs.uk (S.J.); sekhar.marla@uhnm.nhs.uk (S.M.); sankaran.narayanan@uhnm.nhs.uk (S.N.)

**Keywords:** breast reconstruction, implant loss, antibiotic irrigation

## Abstract

*Background and objectives*: Implant-related complications leading to implant loss contribute to major morbidity in immediate breast reconstruction (IBR). Various techniques have been advocated to improve rates of reconstruction salvage. The objective of our study was to assess if a peri-prosthetic irrigation system was an effective adjunct to the conventional wash-out technique in improving reconstruction salvage rates. *Methods*: The study included patients who had immediate implant-based breast reconstruction from January 2015 to November 2020. The conventional technique of reconstruction salvage, using debridement, wash-out, and implant/expander exchange with systemic use of antibiotics, was performed for patients undergoing exploration for infection until May 2019. A simple technique using a continuous peri-prosthetic irrigation system with vancomycin (1 g/L normal saline over 24 h) for 2 days was added as an adjunct to the conventional technique. Treatment details and clinical outcomes were compared between the groups. The study was approved by the Clinical Governance department. *Results*: During the study period, 335 patients underwent IBR. A total of 65 patients (19.4%) returned to the theatre due to post-operative complications, of which 45 (13.4%) were due to infection. A conventional technique was used in 38 (84.4%) patients, and peri-prosthetic irrigation was used as an adjunct in 7 patients (15.6%). A total of 16 (42.1%) in the conventional group and all 7 (100%) in the irrigation group had successful reconstruction salvage. No patients had complications due to antibiotic irrigation. *Conclusions*: The peri-prosthetic irrigation system is a simple, safe, and effective adjunct to conventional techniques in improving reconstruction salvage in IBR.

## 1. Introduction

Breast cancer is the most common cancer affecting women throughout the world, accounting for one in right cancer diagnoses and one in six cancer deaths [1]. The treatment of breast cancer often involves surgery, including breast conservation surgery or mastectomy. Immediate or delayed breast reconstruction is offered along with mastectomy based on choice and suitability. Broadly, there are two types of reconstruction: implant-based and autologous reconstruction. According to the GIRFT (Getting it Right First Time) report, implant-based reconstruction accounts for 45–70% of immediate reconstructions in the UK [2]. Implant reconstruction can be performed immediately as a single-stage—direct implant or two-stage procedure—tissue expander followed by a permanent implant. Immediate breast reconstruction is becoming more common with the introduction of the use of mesh (biological or synthetic) in breast surgery [3]. The use of breast implants can be associated with both short- and long-term complications, including infection, hematoma, seroma, rupture/leak/migration of the implant, and capsular contracture. 

Implant loss is a major complication of immediate breast reconstruction (IBR) and can have a significant impact on overall outcomes, especially patient experience. Implant infection is the most common cause of implant loss. Based on the recent national data and reported studies, 18% of patients returned to the theatre, and 9–10% had implant loss after IBR [4,5]. Following implant loss, the likelihood of delayed reconstruction is only 42% [6]. In addition, patients who proceed with a delayed reconstruction following this complication risk having an inferior aesthetic outcome due to contraction or loss of the skin envelope. The optimal management of peri-prosthetic infection could, therefore, prevent and potentially reduce implant loss, thereby reducing the associated morbidity experienced by patients. Usually, following a diagnosis of implant infection, the initial approach would be to attempt conservative treatment with parenteral antibiotics. If this fails, exploration in the theatre under general anesthesia is undertaken as the next step.

Although various salvage techniques have been reported [6,7,8,9,10], the conventional practice is to explore the wound, followed by debridement and wash-out of the cavity. An exchange to a new implant/expander is performed, and antibiotics are continued in the post-operative period. Even though many other techniques are described in the literature, none of them have been proven superior to the others. Peri-prosthetic irrigation (PPI) is a new technique, and the success of this technique is attributed to the potential reduction in the bacterial count in the area. Burkhardt et al. were the first to describe the intraoperative breast pocket irrigation technique in 1986 for patients undergoing breast augmentation and reported a reduced peri-prosthetic bacterial count and, hence, capsular contracture [11]. Irrigation of wounds and debridement is essential to managing any contaminated wound. Wound irrigation reduces the bacterial count and promotes wound healing [12,13]. In the last few years, we adopted the technique of continuous PPI for implant-related infection following the conventional reconstruction salvage technique. We have described the technique of PPI and compared the outcomes with the conventional technique.

## 2. Methods

The study included patients undergoing mastectomy and immediate implant-based breast reconstruction from January 2015 to November 2020. All patients had routine methicillin-resistant *Staphylococcus aureus* (MRSA) screening and obtained eradication treatment in case of MRSA positivity. All patients underwent pre-pectoral breast reconstruction with an anatomical implant and acellular dermal matrix (ADM). Skin or nipple-sparing mastectomy, with or without an axillary procedure, was performed through a periareolar, inframammary, or skin-reducing incision. The implant was covered completely by the ADM using absorbable sutures. The ADM was fixed to the pectoralis major at three points (3, 9 and 12 ‘0’ clock positions). A single drain was placed routinely in all patients. All procedures were performed as a day case or 24 h stay procedures. Patients were discharged from the hospital with oral antibiotics. Based on the hospital antimicrobial policy, these patients received co-amoxiclav 625 mg or clarithromycin 500 mg BD in case of allergy to penicillin. These patients were regularly followed up in the clinic every week for 4 weeks. Drains were removed when the output was <40 mL/24 h. Patients who developed redness of the breast during the follow-up would have an ultrasound scan of the breast to look for any collection and would undergo aspiration of the collection under aseptic precautions if indicated. The aspirated fluid was sent for culture and sensitivity. Empirical antibiotics were usually commenced until the culture and sensitivity report became available. Patients who developed suspected clinical infections were followed up in an outpatient clinic twice weekly or admitted for intravenous antibiotics if indicated. If not responding to oral antibiotics, patients had a repeat ultrasound of the breast to look for any new collections in the breast pocket. Repeat aspirations were performed if indicated. Surgical exploration was planned if there was no clinical response to antibiotics. A significant majority of patients did not present with systemic signs of sepsis-like fever or chills, as the changes were very local. However, most of them had slightly raised CRP and white cell counts. However, there was no significant difference between the groups in this regard, although the numbers in the PPI cohort were small. 

The goal was to attempt reconstruction salvage. Patients were admitted to the hospital, and intravenous antibiotics were started based on sensitivity and guidance from microbiology. Patients were taken for surgical exploration on the next available emergency theatre slot. Two different techniques were used for salvage during this period. The conventional technique was performed as standard until May 2019. After that, we added PPI as a routine for all patients in addition to the conventional technique. Patients who needed readmission and surgical intervention stayed up to 72 h as an inpatient. The techniques are described below. The demographic and surgical data of patients who developed breast infections were collected from the hospital database, and clinical outcomes were compared between the two groups. This project was registered with the local hospital clinical governance department and obtained approval (UHNMID/CA30023).

Conventional technique:

All patients were given intravenous antibiotics at induction. The same incision used for the nipple-sparing/skin-sparing mastectomy was utilized for exploration of the mastectomy cavity. After implant removal, pus/fluid/tissue was sent for microscopy, culture, and sensitivities. Debridement of the cavity was performed thoroughly with the removal of ADM. After this, the breast pocket was washed with normal saline before inserting a new implant or expander with the use of a new ADM if indicated. Usually, one or two drains were placed based on the size of the cavity. Antibiotics were continued for at least 2 weeks, and an antibiotic prescription was guided by microbiological advice. Patients were usually discharged after 24 h and followed up in the clinic twice weekly for the first two weeks. Drains were monitored and removed once the output was <40 mL/24 h. However, on monitoring, if there was evidence of infection clinically and biochemically, then re-exploration of the cavity was performed with a removal of the implant. Usually, only one attempt at implant salvage was undertaken.

**Figure 1:**
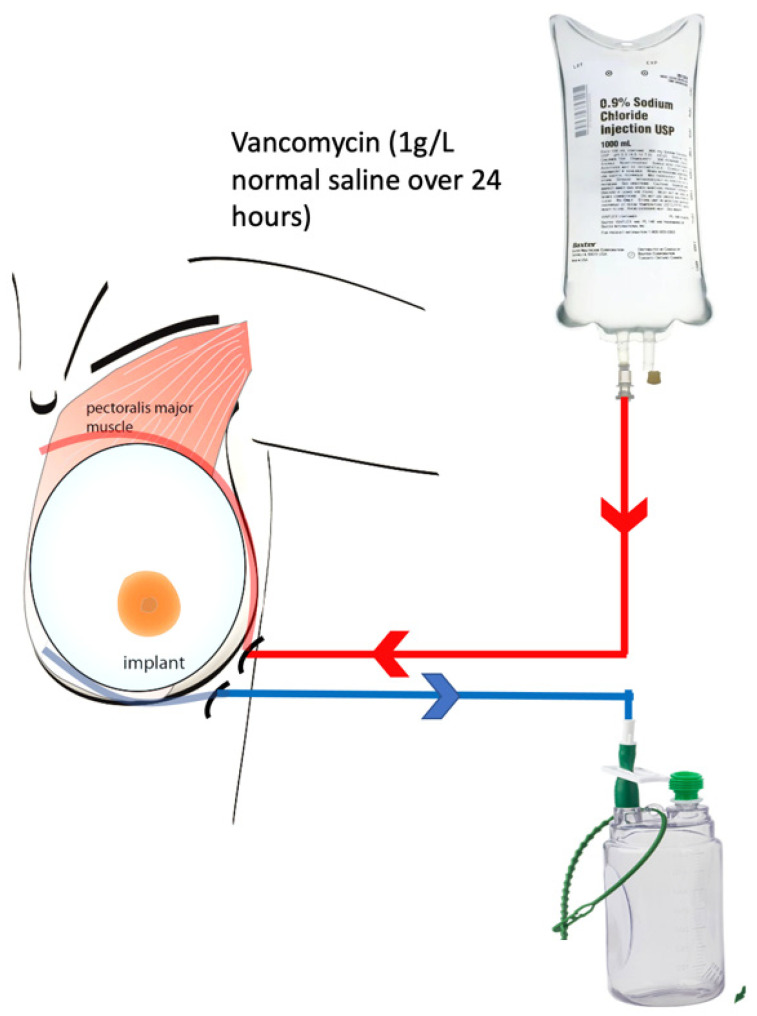
Diagram showing the peri-prosthetic irrigation (PPI) technique. The red line indicates the inflow of antibiotic solution. The blue line indicates the outflow of the irrigation fluid.

Peri-prosthetic irrigation technique (PPI):

The PPI involved using a second drain to irrigate the cavity following the conventional technique. Following the wash-out, debridement, and implant/expander exchange, two drains were placed in the cavity. One drain was placed superior to the implant between the skin and the ADM, which was used as an inflow conduit. Another drain was placed inferior to the implant in the subcutaneous space and used as an outflow drain. The inflow drain was connected to the antibiotic solution with a rate-controlled infuser. The outflow drain was connected to a standard suction drain bottle (Figure 1). Although the ADM mesh was draped around the implant with a few interrupted PDS sutures, it was not a watertight closure, and therefore, it was very likely that the surface of the implant and the mesh would be bathed in the irrigation fluid. We used vancomycin 1 g mixed in 1 L of normal saline to be infused over 24 h. After 24 h, this cycle was repeated for another 24 h. In total, vancomycin 2 g/2 L normal saline was used over 48 h. Instructions were given to the ward nurses for the drain bottle to be changed every 8 h to avoid overfilling and overflow problems. Serum vancomycin levels were checked after 48 h. Vancomycin was chosen as the antibiotic for irrigation based on the advice from the microbiologists. Microbiologists advised using vancomycin based on the most common bacteria isolated (*Staphylococcus aureus*) and sensitivity pattern. Empirical systemic antibiotics were continued for 48 h, and antibiotics were changed if necessary based on culture and sensitivity reports. The superiorly placed inflow drain was removed after 48 h, and the inferior outflow drain was used as the standard drain and removed when the output was reduced to <40 mL/24 h. Patients who underwent PPI were usually discharged home after 72 h and followed up in the clinic twice weekly for the first 2 weeks. They were evaluated clinically via symptom assessment and clinical examination. An ultrasound scan was performed if there were any signs of persistent redness or seroma. Patients were usually continued on oral antibiotics until the drains were removed and followed up in the clinic for 4 weeks. 

Statistical analysis: Quantitative data like age and BMI were represented with central tendency measures and analyzed using the Student *t*-test. Qualitative data were analyzed using the chi-square test. *p*-values of <0.05 were considered to indicate statistical significance.

## 3. Results

During the study period, 335 patients underwent IBR. A total of 65 patients (19.4%) returned to the theatre due to post-operative complications, of which 45 (13.4%) were due to infection. A conventional technique was used in 38 (84.4%) patients, and PPI was used as an adjunct in 7 patients (15.6%). Demographics and clinical characteristics are presented in Table 1. There was no difference between the two groups with respect to age, smoking status, BMI, and type of surgery. A total of 16 (42.1%) in the conventional group and all 7 (100%) in the irrigation group had successful reconstruction salvage. There were no complications due to antibiotic irrigation. All patients usually stayed in the hospital for an average of three days post-operatively and were then discharged home with oral antibiotics. The patients who eventually failed salvage usually presented with ongoing issues in the next couple of weeks. There was no difference between the groups in terms of the use of expanders. In general, most of the cultures taken from the pus did not show any organisms, as many patients were prescribed empirical antibiotics on presentation. 

## 4. Discussion

Our study demonstrates a 100% success rate (7/7) with the addition of the PPI system to the conventional technique for reconstruction salvage in immediate implant-based breast reconstruction. PPI seems to be an effective adjunct to conventional techniques and is not associated with any complications with its use. There were no specific differences in hospital stay, and adding another drain to the wound in the theatre did not significantly impact the duration of the operation. 

Various techniques have been described in the literature for implant salvage with variable success rates (Table 2) [6,7,8,9,10]. A similar technique was described by Ngi-wieh Yii et al., who reported 64% successful implant salvage [7]. The implant cavity was reopened and scrubbed with povidone-iodine. The cavity was then irrigated with half-strength hydrogen peroxide, followed by normal saline. A capsulotomy was performed to release tension on the flaps, and two drains were placed, one for inflow of irrigation fluid and one for outflow. A new implant was inserted, and the cavity closed. Normal saline irrigation was started immediately, and 1 g of flucloxacillin was administered intermittently every 6 h. This was continued for 5 days. Most patients stayed in the hospital for 5–7 days and received intravenous antibiotics [7]. Compared to our technique, although capsulectomy was performed and the hospital stay was longer, salvage success rates were lower. 

Aggressive debridement techniques have also been reported with success in implant salvage. Prince et al. attempted implant salvage in 43 patients by performing aggressive curettage of the implant pocket until the tissue bled, followed by 6 L of pulse lavage irrigation (3 L normal saline and 3 L antibiotic solution) [8]. After lavage, a new implant was inserted, and a 15-French drain was placed. Antibiotics were given for 4–6 weeks, according to culture and sensitivity reports. With this protocol, 33 patients had successful implant salvage (76.74%) [8]. These techniques have similarities with our technique; however, the success rate of salvage was higher in our series, and only 48 h of irrigation after surgery was used, along with a shorter hospital stay.

Implant pocket curettage and pulse lavage with copious amounts of saline have been used in multiple studies with varying results. Chun et al. reported a 100% (9/9) success rate with their technique of implant salvage [9]. All patients received vancomycin intravenously until operative intervention. The surgical technique involved the curettage of fibrinous and infectious material using a sponge stick. The pocket was irrigated with 6 to 9 L of normal saline by pulse lavage. No formal capsulectomy was performed. A new implant of the same size was inserted, and two drains were placed. The patients remained on intravenous antibiotics during the immediate post-operative period while awaiting microbiologic results. Patients were given post-operative antibiotics for 4 weeks and stayed in the hospital for 3–11 days [9]. However, another study utilizing similar techniques reported only a 45% (9/20) successful implant salvage rate in their series, which was attempted only in mild infections. They treated all patients with intravenous antibiotics, wound debridement, capsulotomy, saline lavage, new implant placement, and closed suction drain [6]. Most of these salvage techniques have stressed copious lavage and had lower success rates with severe peri-prosthetic infection. 

The decision making about the timing of surgical intervention can have an impact on the success rates in implant salvage. In our experience, we have learned that early intervention can translate to successful implant salvage. Sarfati et al. from the Paris Breast Centre published retrospective data of 80 patients who developed implant infection [14]. In their institution, all patients received oral antibiotics and were reviewed by the same surgeon every 2 days. If there was improvement, antibiotics were continued for 3–10 weeks. Surgery was considered in cases of failed medical management. Surgery included removal of the implant, microbiological sampling of fluid, wash-out of pocket with betadine and saline, placement of a drain, and exchange to a new implant of the same size. They reported an 88.8% (71 patients) successful salvage using this technique. An important point to note from their study was that only 18 patients (22.5%) required surgery for failed conservative therapy with antibiotics, and among them, 10 patients (55.55%) had successful implant salvage. Three patients underwent surgery due to features of sepsis, and of them, two (66.67%) had successful salvage [14]. The lower rate of salvage in the number of patients who underwent surgical intervention seems to suggest that slightly earlier intervention could have a positive impact on salvage rates, but this can be difficult to predict. The reasons for implant loss are multifactorial, and therefore, decision making regarding the timing of intervention can be difficult.

Multiple innovative methods have also been reported in the literature with the use of antibiotic plates, negative pressure systems, and Pulsavac systems. Albright et al., from Houston, Texas, used antibiotic plates and reported 100% successful implant salvage between January 2009 and February 2014 [15]. The salvage technique involved thorough surgical debridement with the removal of the acellular dermal matrix and wash of the pocket with 3 L of ringer lactate mixed with 50,000 U of bacitracin. The new implant was soaked in a triple-antibiotic solution (1 g vancomycin, 80 mg gentamicin, and 50,000 U bacitracin. The antibiotic plates were created with one package of antibiotic-loaded polymethylmethacrylate (PMMA), which includes 2.2 g of tobramycin and 4 g of vancomycin. The antibiotic plates measured 0.2 cm × 0.5 cm in thickness and were contoured to the chest wall. The PMMA plate was then placed in the pocket between the chest wall and the implant. Prior to closure, one or two closed-suction drains were placed in the pocket to allow for serial drain fluid cultures. They performed this procedure on 14 patients with a 100% success rate and no complications [15]. 

The use of negative pressure systems has always shown high success rates in wound healing, and a technique of implant salvage using negative pressure wound therapy (NPWT) after removal of prosthesis [16] was reported from Australia. NPWT used the instillation of a topical solution of 100 to 150 mL into the breast pocket and 15 min soaking time. After 15 min, the fluid was suctioned for 3.5 h at −125 mm of Hg sub-atmospheric pressure. The device used is called V.A.C. VeraFlo Therapy (Kinetic Concepts, Inc., San Antonio, TX, USA). All patients received antibiotics for at least 3 weeks. The new implant was restored after infection control. They reported an 83% success rate with this technique (23/28 patients) [16]. Lauren E. Antognoli et al. from the United States (US) used V.A.C. VeraFlo in 16 patients and salvaged implants in 15 patients with a success rate of 93.75%. On top of this, the use of Veraflo was cost-effective compared to other techniques [17]. 

Marcasciano et al. used pulse therapy using Pulsavac (Pulsavac Plus, Zimmer Surgical Inc., Dover, OH, USA) to cleanse the surface of the breast pocket before replacing the implant [18]. Pulsavac produces intermittent, pulsed irrigation of the peri-prosthetic tissues that prevents the formation of biofilm and, hence, treats infection by reducing bacterial load. They tried this technique in eight patients and successfully salvaged all of them [18]. In all of these innovative techniques, success rates are high; however, it involves the use of complex kits and multiple antibiotics. 

Staged procedures have also been shown to have promising results. Muthuswami et al. from the UK admitted all patients with implant-related breast infections to the hospital for 5 days of intravenous antibiotics [19]. At the end of the 5 days, if there was no response, and the patients were taken for surgical salvage. The surgical technique involved the removal of the implant and filling the cavity with a solution of gentamicin and saline. After 48 h, the cavity was explored under GA, and a new implant was inserted. With this technique, they were able to salvage all five patients who developed breast implant infections between 2017 and 2019 [19]. In a similar way, Oren Lapid removed the implant and cleaned the pockets with pulse lavage [20]. The implant was replaced with a gentamicin collagen sponge. This sponge achieves a high local concentration of antibiotics and hence treats the infection. With this technique, the author reported successful salvage in four patients [20]. However, in both of these approaches, there is a need for multiple operations and longer hospital stays.

Some series have shown high success rates with a single-stage washing of the cavity and antibiotics. H Yeo et al. from Korea reported an infection rate of 5.5% (8/145) after implant-based reconstruction. After removing the implant, a thorough wash of the cavity with povidone-iodine and antibiotics was performed. A new implant was placed, and a drain was inserted in all patients. With this technique, they reported a success rate of 87.5% [21]. Although the number of surgical interventions is small, it raises the possibility that the probable timing of intervention may have a potential impact on salvage rates.

Implant salvage using similar techniques has been reported in breast augmentations as well. M Sforza reported their technique of salvage in augmentation, which included removal of the implant, cleaning of the cavity, and immediate insertion of a new prosthesis and drain. All patients received intravenous antibiotics post-operatively. A total of 17 patients developed infection, and 3012 patients underwent primary breast augmentation. Of these 17 patients, everyone had successful implant salvage with an antibiotic scrub and insertion of a new implant [22]. The high salvage rates in these series involving augmentations are difficult to compare to immediate breast reconstructions, considering the difference in vascularity of the area and the thickness of the flaps. 

Many centers in the UK and worldwide explant the prosthesis without any effort to salvage the implant in case of severe breast infections. This management principle comes from the guidelines for sepsis control: Sepsis Six with source control [23]. This approach is not entirely appropriate for breast implant infection with a subsequent physical and psychological impact on the patient [24]. Delayed reconstruction after infection control also poses a challenge to the surgeon in terms of choice of reconstruction. Due to skin fibrosis and loss of muscle bulk, these patients may require autologous flap reconstruction. Successful salvage maintains the breast mound and the skin envelope, allowing women to have the option of fat grafting and capsulotomy with an exchange implant in the future in cases of capsular contracture. 

Our technique, with the addition of a simple PPI system to the conventional technique, has demonstrated higher success in salvage rates. However, higher numbers in the group would be useful for significance purposes. Central to our protocol is continuous wound/cavity irrigation, which is likely to reduce bacterial load and promote wound healing [12,13]. The added advantages are the simplicity of the technique and the lack of need for any specialized equipment or kit to deliver the irrigation. 

## 5. Conclusions

The peri-prosthetic irrigation system is a simple, safe, and effective technique in improving implant/reconstruction salvage in IBR. PPI does not need any special equipment apart from the infusion pump, and there is no specific impact on the duration of hospital stay. 

## Figures and Tables

**Table 1:** Demographics and clinical characteristics.

Characteristic	Conventional Group (n = 38)	Peri-Prosthetic Irrigation Group (n = 7)	*p*-Value
Age			
1. Mean	55	53	0.423
2. Median	54	54	
3. Range	37–68	44–62	
Smoking status			
1. Yes	2	1	0.379
2. No	36	6	
BMI			
1. Mean	28	30	0.264
2. Median	31	31	
Indication for mastectomy			
1. Invasive cancer	23	4	0.961
2. Ductal carcinoma in situ	9	2	
3. Risk-reducing mastectomy	6	1	
Type of mastectomy			
1. Skin sparing mastectomy	30	4	0.441
2. Nipple-sparing mastectomy	6	2	
3. Skin reducing mastectomy	2	1	
Axillary surgery			
1. Sentinel lymph node biopsy	28	4	0.431
2. Axillary nodal clearance	4	2	
3. None	6	1	
Successful Implant salvage	16	7	0.019

**Table 2:** Various studies on reconstruction salvage techniques.

Sl No.	Authors	Year	Salvage Technique	Success Rate
1	Our study	2023	Peri-prosthetic irrigation	7/7 (100%)
2	Ngi-Wieh Yii [7]	2003	Capsule cavity scrubbed with iodine, then half-strength peroxide, followed by copious saline wash, capsulotomy, and implant exchange. Two drains, wound closed. Continuous saline and intermittent antibiotic irrigation through the drains.	9/14 (64.28%)
3	Prince MD et al. [8]	2012	Broad spectrum antibiotics+ Early operation-curettage of necrotic tissue + Pulse lavage with 3L NS and 3L Antibiotics + Implant exchange	33/43 (76.74%)
4	Jin K Chun [9]	2007	Intravenous antibiotics followed by drainage of fluid, manual debridement and curettage of the infected pocket, device exchange, and post-operative antibiotics.	9/9 (100%)
5	Bennett et al. [6]	2011	Debridement + Capsulotomy +saline wash and either: autologous flap + implant; reinsertion of same size; smaller implant; advancement flap.	9/20 (45%)
6	Richard G Reish et al. [10]	2013	Intravenous antibiotics and implant exchange	37/99 (37.3%)
7	I Sarfati et al. [14]	2020	Medical management with antibiotics Surgical management with wash-out and exchange of implant	71/80 (88.8%)
8	S B Albright et al. [15]	2016	Debridement and antibiotic-loaded polymethylmethacrylate (PMMA) sheets and systemic antibiotics	14/14 (100%)
9	F Meybodi et al. [16]	2021	Explant + negative pressure wound therapy(NPWT)/(V.A.C. VERAFLO).	23/28 (83%)
10	Lauren E. Antognoli et al. [17]	2021	Explantation + debridement + wash-out of the breast pocket NPWT (V.A.C. VERAFLO) with Prontosan as an instillation solution.	15/16 (93.75%)
11	M Marcasciano et al. [18]	2022	Pulse therapy using pulsavac	8/5 (100%)
12	A Muthusami et al. [19]	2020	Removal of the implant and filling the cavity with a solution of gentamicin and saline. After 48 h, the cavity is explored, and a new implant is inserted.	5/5 (100%)
13	O Lapid et al [20]	2011	Gentamicin collagen sponge	4/4 (100%)
14	H Yeo et al. [21]	2021	Debridement + povidone-iodine/antibiotic lavage in the implant pocket + implant replacement	7/8 (87.5%)
15	M Sforza et al. [22]	2014	Explant + Capsulotomy + Antibiotic scrub + Insertion of a new implant	17/17 (100%)

## Data Availability

The data presented in this study are available on request from the corresponding author. The data are not publicly available due to General data protection regulation.
